# Dalbavancin treatment in a deep sternal wound MRSA infection after coronary artery bypass surgery: a case report

**DOI:** 10.1186/s13019-017-0690-5

**Published:** 2018-01-05

**Authors:** Aneta GUZEK, Grzegorz SUWALSKI, Dariusz TOMASZEWSKI, Zbigniew RYBICKI

**Affiliations:** 1 Department of Microbiology, Military Institute of Medicine, Warsaw, Poland; 2 Department of Heart Surgery, Military Institute of Medicine, Warsaw, Poland; 3 Department of Anesthesiology and Intensive Therapy, Military Institute of Medicine, ul. Szaserów 128, 04-141 Warsaw, Poland

**Keywords:** Deep sternal wound infections, Dalbavancin, Vancomycin, Methicillin-resistant *Staphylococcus aureus*

## Abstract

**Background:**

A deep sternal wound infection (DSWI) can become a severe complication after cardiac surgery, with in-hospital mortality rates reaching up to 35%. Staphylococci, particularly methicillin resistant *Staphylococcus aureus* (MRSA), play important roles in its etiology.

**Case Presentation:**

This case report presents a patient who underwent coronary artery bypass surgery, and suffered postoperatively from a DSWI caused by MRSA. The pathogen was susceptible to vancomycin and rifampicin in vitro; however, this therapy was clinically ineffective. Both clinical improvement and MRSA eradication were achieved after surgical debridement of the wound and the intravenous administration of dalbavancin.

**Conclusions:**

We decided to administer dalbavancin because of its convenient pharmacological profile. The patient’s tolerance of the antimicrobial was good, the biochemical markers of inflammation returned to the normal ranges, and the microbiological results one week after the dalbavancin administration were negative. A good clinical outcome was achieved with both the surgery and antimicrobial administration. In this case, dalbavancin was more effective in the treatment of the sternal and surrounding tissue infections caused by MRSA, when compared to vancomycin.

## Background

One possibly severe complication after cardiac surgery is a deep sternal wound infection (DSWI). Its incidence varies from 0.5% to 6.8%, with an in-hospital mortality rate ranging between 7% and 35% [1]. Staphylococci, including methicillin-resistant *Staphylococcus aureus* (MRSA), are the most common etiological factors of these wound infections [1, 2]. In patients undergoing sternotomies, the sternum is often the site of infection because of its decreased vascularity from the surgical procedure. Moreover, DSWIs are potentially devastating complications that are difficult to treat when compared to skin and subcutaneous tissue infections. Fortunately, the systemic administration of vancomycin improves the clinical situation in the majority of cases.

## Case presentation

Here we report the case of a 60-year old obese male patient, with severe cardiac problems, chronic obstructive pulmonary disease, hypertension, insulin dependent diabetes mellitus, and arteriosclerotic vascular disease. This patient underwent heart surgery on July 17, 2016, four days after an ST-elevation myocardial infarction following pulmonary edema. During the procedure, a complete arterial revascularization was performed (LIMA^1^ to LADA,^2^ LRA^3^ → LIMA /Y graft/ to OMA,^4^ RIMA^5^ to RCA,^6^ LRA → RIMA /Y graft/ to PDA^7^). On September 11, 2016, this patient was admitted to the Department of Cardiology of the Military Institute of Medicine in Warsaw, Poland due to severe circulatory failure [New York Heart Association (NYHA) class III, N-terminal pro b-type natriuretic peptide (NT-proBNP) serum concentration = 2987 pg/mL]. His body temperature was 38 °C. He had a purulent wound in the lower part of his sternum, with a fistula approximately 2 cm long. There were also signs of inflammation in the surrounding tissues, and the middle section of his sternum was moderately unstable. His laboratory tests revealed an increased number of white blood cells (15.5 × 109/L), and his C–reactive protein serum level was 34.9 mg/dL.

Specimens were collected from the wound, blood, and urine for the microbiological analyses. Microbiological identification was performed with VITEK2 analyzer (BioMérieux, France), according to the manufacturer’s guidelines. Minimal inhibitory concentration (MIC) values for different antimicrobial agents were determined with Etest (BioMérieux, France). Because the isolated pathogen was susceptible for vancomycin, such antimicrobial agent (1 g every 12 h) was started. A computed tomography scan of his thorax showed no signs of mediastinitis, and the blood and urine samples were microbiologically negative. MSRA was isolated from the wound, and this pathogen was susceptible to vancomycin (MIC = 0.75 mg/L), rifampicin, gentamycin, teicoplanin, daptomycin, linezolid, tigecycline, trimethoprim/sulfamethoxazole, and dalbavancin (MIC = 0.064 mg/L) (Table 1).

**Table 1:** Microbiological analysis of the sample taken from the sternal wound. Identified pathogen: methicillin resistant *Staphylococcus aureus* (MRSA)

antimicrobial	MIC (mg/L)	interpretation	breakpoints
vancomycin	1	S	2; 2
ciprofloxacin	> 4	R	1; 1
gentamycin	4	R	1; 1
rifampicin	≤ 0.03	S	0.06; 0.5
daptomycin	0.5	S	1; 1
linezolid	1	S	4; 4
dalbavancin	0.064	S	0.125; 0.125
tigecycline	0.25	S	0.5; 0.5
TMT/SMX	> 160	R	2; 4

S - susceptible, R - resistant

Although the serum concentration of the vancomycin was within the therapeutic range (10–15 mg/L), we observed lacks of both local and clinical improvement. In this situation, rifampicin (300 mg every 12 h) was added to the vancomycin; however, the patient still complained about the pain during breathing and chest palpation. Purulent secretions from the wound were also observed. Therefore, a decision was made to administer dalbavancin (1500 mg intravenously), with refixation of the sternum. Surgery was performed on September 22, 2016, after an improvement in this patient’s general condition.

**Fig. 1:**
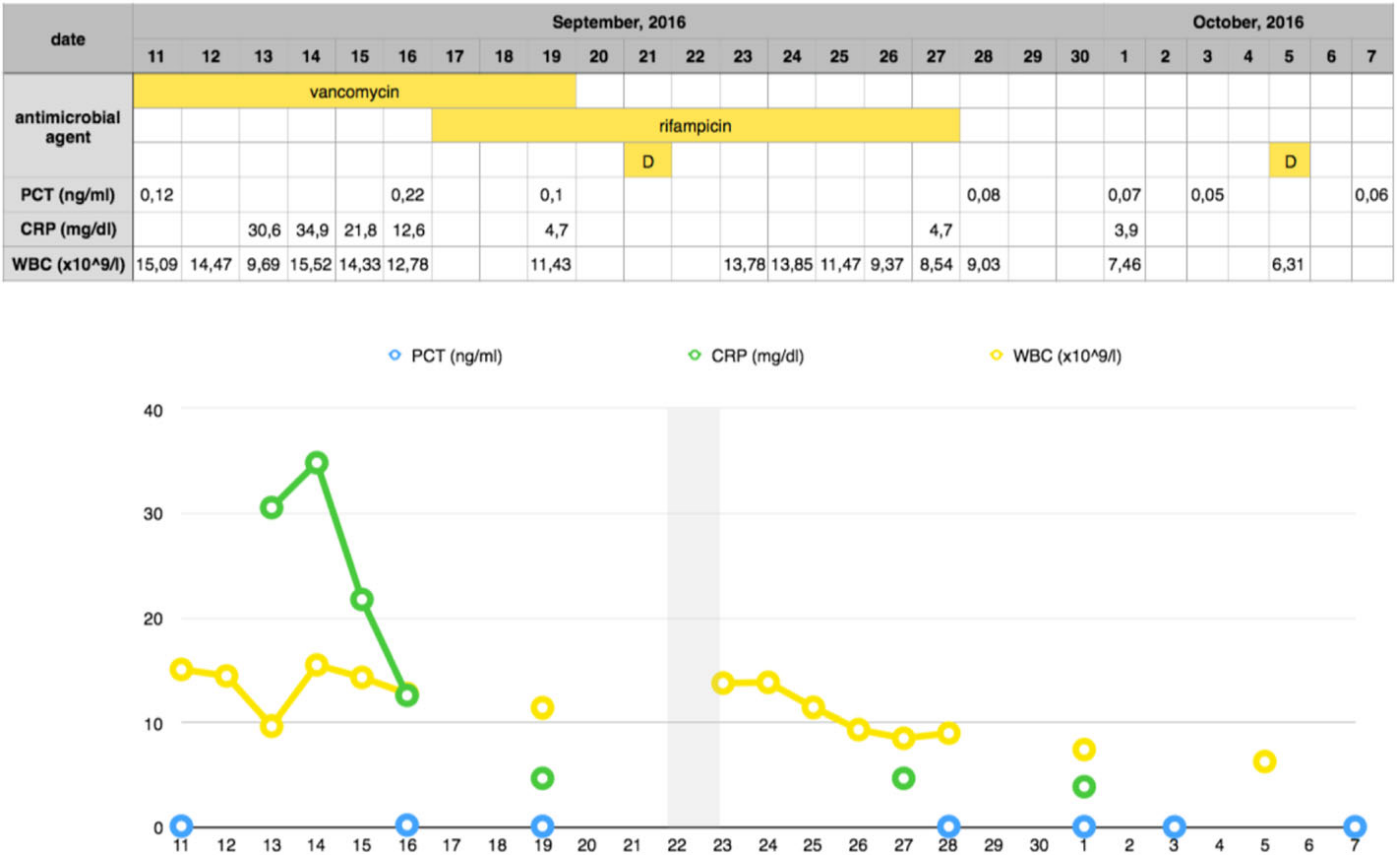
Changes in the serum concentrations of the inflammatory markers

Intraoperatively, there were signs of mediastinitis, with a few pus reservoirs under the sternum. Impaired wound healing with a separation of the sternum was diagnosed. The necrotic tissues were removed, the mediastinum was rinsed with antiseptic, and irrigated drainage with gentamycin was started. A second dose of dalbavancin (1500 mg) was administered after 14 days of the therapy. Overall, the postoperative period was uncomplicated, with normal wound healing observed, as well as improvement in the circulatory function and a decrease in the serum inflammatory markers. On September 28, 2016, after seven days of dalbavancin therapy, a microbiological examination of the removed drains did not reveal the pathogens. The changes in the serum concentrations of the biochemical markers of inflammation are presented in Fig. 1.

This patient was discharged in good local and general condition on October 7, 2016, fifteen days after the surgery.

## Discussion and conclusions

The patient in the case presented above was diagnosed with a DSWI. In general, bilateral internal mammary artery grafts, diabetes, and male sex are predictors of such a complication [3].

The serum concentrations of procalcitonin suggested that it was a local infection. Although the vancomycin was microbiologically effective against the MRSA organisms isolated from the wound, and the use of vancomycin did result in reduction in CRP values over time, we observed no signs of local or clinical improvement. This lack of effect could be due to relatively poor penetration of vancomycin into the bones [4]. In addition, vancomycin is a bacteriostatic rather than bactericidal agent; in a case such as this, this bacteriostatic property could have played a crucial role. Although vancomycin has been administered for nearly 60 years, it is still mentioned in the recommendations of many scientific societies, such as the Infectious Diseases Society of America (IDSA) [5]. The therapy of osteomyelitis is long-lasting process, not shorter than 8 weeks, so vancomycin may be administered only at the beginning of this process. When the MIC value of vancomycin increases, the higher doses of antimicrobial are required to achieve a necessary serum concentration of the drug (between 20 and 25 mg/L), especially in patients who suffer from severe infections. Unfortunately, the nephrotoxicity of vancomycin also increases. Moreover, the clinical outcome depends on the MIC value of antimicrobial agent, as well as the probability of recurrent infection [6, 7]. In the described case, the MIC value of vancomycin was 0.75 mg/L, which was close to ‘creep’ value. This means that the MIC was below the breakpoint; although the clinical efficacy of the antimicrobial could have been insufficient. In such cases, the administration of different antibiotic is suggested [8]. The problem of nephrotoxicity is an important factor in patients who have undergone cardiac surgery procedures, where the incidence of nephrotoxicity can reach 30% [9]. In this situation, we decided to administer dalbavancin, a bactericidal antibiotic with long-lasting antimicrobial activity (up to 14 days after a single intravenous administration). Because of its relatively low systemic toxicity and its good tissue penetration (including to bones), dalbavancin was approved in the European Union for the treatment of acute bacterial skin and skin structure infections in 2016. Although new cephalosporins with anti-MRSA activity, such as ceftaroline and ceftobiprole, exhibit similar activity against Gram-positive cocci [10], we chose an antimicrobial with a more convenient pharmacological profile and non-inferior efficacy when compared to vancomycin [11, 12]. For example, Jones et al. [13] found that dalbavancin was 16-fold more active against *Streptococcus pneumoniae* than vancomycin (MIC50 = 0.015 vs. 0.25 mcg/ml, respectively). According to Boucher et al., clinical success was observed in 90.6% of the patients infected with *S. aureus*, including MRSA, treated with dalbavancin [11].

This antimicrobial was administered twice in the described case. Such administration should provide tissue exposure over the dalbavancin MIC for *Staphylococcus aureus* for 8 weeks [14, 15], and may show clinical benefits in the treatment of osteomyelitis [15]. However, newer studies suggest that dalbavancin may be administered in single dose, with non-inferior efficacy when compared to the two-dose administration [16, 17]. The patient was discharged home after the administration of the second dose of 1500 mg of dalbavancin, which improved both his comfort level and decreased the costs of hospitalization. In the presented case, the patient’s tolerance of the antimicrobial was good, with no adverse reactions observed. The biochemical markers of inflammation returned to the normal ranges, and the microbiological results one week after the dalbavancin administration were negative. A good clinical outcome was achieved with both the surgery and the antimicrobial administration. One month after the surgery patient’s wound was completely healed and his sternum was stable. In this case, the dalbavancin was more effective than the vancomycin due to its bactericidal action, and better penetration into the bones. Moreover, the MIC of the dalbavancin was much lower than that of the vancomycin, despite the fact that the latter was below the breakpoint value.

In this case, dalbavancin was more effective in the treatment of the sternal and surrounding tissue infections caused by MRSA, when compared to vancomycin. Dalbavancin may be an interesting therapeutic choice when administration of vancomycin is clinically inefficient or contraindicated. Dalbavancin also may be an alternative for long-lasting therapy of osteomyelitis, when administration of vancomycin and linezolid are out of option. In such cases therapy with trimethoprim/sulfamethoxazole is the only choice, because of good susceptibility of MRSA strains for this antimicrobial agent. This case report supports a definitive clinical trial of dalbavancin for the treatment of osteomyelitis.

## Abbreviations

LADA: Left anterior descending artery
LIMA: Left internal mammary artery
LRA: Left radial artery
MRSA: Methicillin-resistant *Staphylococcus aureus*
OMA: Obtuse marginal artery
PDA: Posterior descending artery
RCA: Right coronary artery
RIMA: Right internal mammary artery

## Footnotes

1 LIMA – left internal mammary artery

2 LADA – left anterior descending artery

3 LRA – left radial artery

4 OMA – obtuse marginal artery

5 RIMA – right internal mammary artery

6 RCA – right coronary artery

7 PDA – posterior descending artery
